# Flash (Ultra-Rapid) Spark-Plasma Sintering of Silicon Carbide

**DOI:** 10.1038/srep33408

**Published:** 2016-09-14

**Authors:** Eugene A. Olevsky, Stephen M. Rolfing, Andrey L. Maximenko

**Affiliations:** 1 San Diego State University, San Diego, CA, USA

## Abstract

A new ultra-rapid process of flash spark plasma sintering is developed. The idea of flash spark plasma sintering (or flash hot pressing - FHP) stems from the conducted theoretical analysis of the role of thermal runaway phenomena for material processing by flash sintering. The major purpose of the present study is to theoretically analyze the thermal runaway nature of flash sintering and to experimentally address the challenge of uncontrollable thermal conditions by the stabilization of the flash sintering process through the application of the external pressure. The effectiveness of the developed FHP technique is demonstrated by the few seconds–long consolidation of SiC powder in an industrial spark plasma sintering device. Specially designed sacrificial dies heat the pre-compacted SiC powder specimens to a critical temperature before applying any voltage to the powder volume and allowing the electrode-punches of the SPS device setup to contact the specimens and pass electric current through them under elevated temperatures. The experimental results demonstrate that flash sintering phenomena can be realized using conventional SPS devices. The usage of hybrid heating SPS devices is pointed out as the mainstream direction for the future studies and utilization of the new flash hot pressing (ultra-rapid spark plasma sintering) technique.

Electromagnetic field-assisted sintering techniques have increasingly attracted attention of scientists and technologists over the course of the last decade^1^,^2^,^3^,^4^,^5^,^6^,^7^,^8^,^9^,^10^,^11^,^12^,^13^,^14^. Spark-plasma sintering (SPS) is a particular kind of field-assisted sintering, which significantly shortens processing of powder materials and improves the powder consolidation performance in terms of both time and quality. It is especially promising with regard to maintaining the nano and sub-micron structure in nano-powder-based materials after consolidation. SPS gains particular prominence in connection with its unique capabilities of processing very hard-to-deform materials, which would typically require lengthy consolidation times at significantly elevated temperatures under conditions of conventional powder pressing or sintering.

Another field-assisted phenomenon - the “flash sintering”^15^,^16^,^17^,^18^,^19^,^20^,^21^,^22^,^23^,^24^,^25^,^26^,^27^,^28^,^29^,^30^,^31^,^32^,^33^,^34^, which has been recently explored by a number of researchers, often enables ultra-rapid densification (in a matter of seconds) of ceramic specimens (mostly oxides, and some carbides). In the traditional flash sintering setup the powder specimen is placed in a tube furnace with two electrodes attached. The specimen is heated to a certain critical level of temperature, and then the electric current passes through it based on a DC power supply, which is connected to the specimen by the above-mentioned electrodes. The applied voltage is usually of the order of 50–150 volts or higher, which places flash sintering in the higher (compared to SPS) voltage range among the field-assisted sintering techniques. SPS is normally conducted under lower voltages (less than 10 V).

Some other ultra-rapid sintering techniques have been explored in the past. For example, plasma-assisted sintering (also assisted by microwaves) triggered an intense discussion and attracted significant interest in 1980s^35^,^36^. Under plasma sintering conditions, small specimens could be sintered during several seconds only. One of the major reasons of why this approach didn’t find broad applications is the non-uniformity of the internal heating of the processed specimens, causing the heterogeneity and damage (due to thermal shock-like conditions) of the specimen’s structure and significant difficulty of the process scalability. Similar problems should be expected also for the flash sintering technique. Indeed, despite some alternative explanations^15^,^16^,^18^,^21^,^22^,^23^,^26^,^28^, it is highly probably that flash sintering is closely associated with thermal runaway events, taking place in ceramic materials subjected to heating by electric current. It is known that electrical conductivity of many ceramic materials increases under elevated temperatures. Under the voltage-control regime, the increase of the electric current leads to the higher generation of Joule heat, which, in turn, causes even higher increase of the specimen’s temperature, and so on, so forth – the heating process under these conditions assumes the “avalanche” regime known as “thermal runaway”. In recent publications on flash sintering there are strong indications of the substantial difference in the temperatures of the tube furnace and the specimen^26^.

The major purpose of the present study was to theoretically demonstrate the thermal runaway nature of flash sintering and to experimentally address the challenge of un-controllable thermal conditions by the stabilization of the flash sintering process through the application of the external pressure. This led us to the idea of conducting flash sintering inside the device capable of the simultaneous application of an external pressure and of an electric current to the processed specimen under elevated temperatures. One possible solution was the application of the current-assisted processing regime inside an SPS or hot pressing device^37^,^38^. This approach may enable a controllable ultra-rapid processing of ceramic powder materials.

## Results

Our investigations showed that the consolidation efficiency of spark plasma sintering can be dramatically further improved by enabling an ultra-rapid “flash” regime of processing, when super-hard powder materials can be consolidated in a matter of few seconds.

The idea of the “FHP” is based upon the idea of recently explored “flash sintering”^15^,^16^,^17^,^18^,^19^,^20^,^21^,^22^,^23^,^24^,^25^,^26^,^27^,^28^,^29^,^30^,^31^,^32^, however, it does not require the usage of higher voltage ranges, employed by “flash sintering” setups (and, therefore, not achievable in the industrially produced SPS devices.) Hence, FHP can be conducted in regular SPS devices. FHP utilizes the theoretical idea of the thermal-runaway-based origin of the flash sintering phenomenon. As opposed to flash sintering, which should generally render uncontrollable clustered area heating of the processed specimen and therefore should lead to highly spatially non-uniform distribution of temperature, density and of microstructure parameters, FHP explores the possibility of the pressure-controlled thermal runaway, under which the mass transport driven by externally applied pressure should equalize the distribution of temperature and stabilize the process of consolidation. In contrast to flash sintering, which should have drastic scalability problems, FHP has a potential of being employed for large scale specimens, where the uniformity of relative density and grain size is of great importance. At the same time it should be noted that the above-mentioned large-scale specimen applicability of FHP requires further thorough analyses in light of the known scalability problems of the regular SPS processes^9^,^10^.

Conducting flash sintering in a device designed for spark plasma sintering requires extra thought in die design. The goal is to pass current through the die alone, heating the specimen by radiation heating, then switching so that electrical current can only flow through the specimen. The proposed solution is to use a sacrificial conducting collar made out of conductive material. This collar would permit the flow of electrical current from the “top punch” to the “bottom die” only.

**Figure 1:**
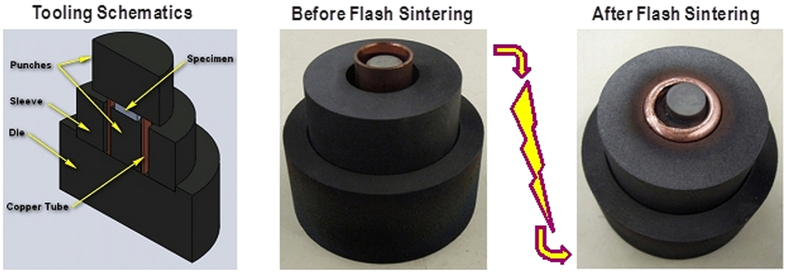
Shown above is the FHP die system. These dies allow the specimen to be isolated from electrical current while being heated through radiation heating. At critical temperature, the copper tube collapses and the specimen is exposed to the current of the SPS device.

The top punch at the start of the experiment is not in contact with the specimen. As the current flows, the collar’s temperature heats up according to the regime programmed into the SPS device at the start of the experiment. In the conducted experiments on the consolidation of SiC powder, a copper tube has been used in the capacity of the sacrificial conducting collar. Copper has a melting point of 1085 °C, and, as the temperature approaches this value, the collar becomes very soft and slumps downwards allowing the top punch to make a contact with the specimen. A scale diagram of the die cross-section is given in Fig. 1.

**Figure 2:**
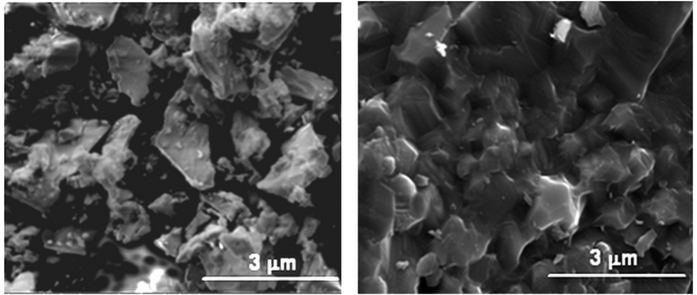
SEM micrograph of SiC powder (left), and SiC specimen processed by FHP (right).

**Figure 3:**
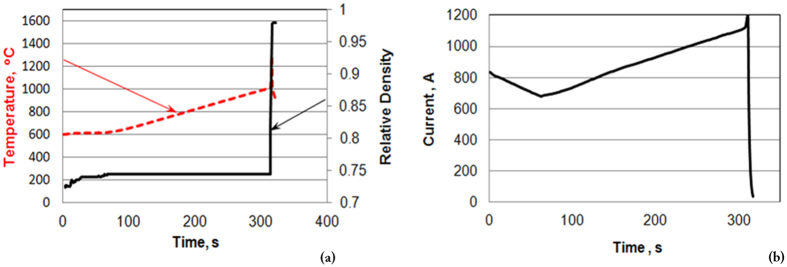
(**a**) Temperature (dashed line) and relative density (solid line) evolution during FHP. A spike in temperature corresponds to the onset of sintering. (**b**) Electric current evolution during FHP.

The results of the FHP processing of a SiC powder are shown in Fig. 2. One can see that (as a result of 1–2 s processing) the SiC specimen has high relative density (99%, with the evidence of the limited grain growth.) The temperature and relative density evolution during FHP are shown in Fig. 3. A 1000 °C spike in temperature (Fig. 3(a)) and electric current (Fig. 3(b)) correspond to the onset of FHP. The data in Fig. 3 provide evidence of the thermal runaway nature of the FHP process.

## Discussion

As mentioned above, the ultra-rapid densification under FHP conditions is associated with the thermal runaway occurring in a powder material under the influence of self-accelerating Joule heating. While for pressure-less flash sintering the thermal runaway should have an un-controllable character leading to substantial non-uniformity of temperature, and in turn, of density and of other structure parameters’ distributions in the processed powder volume (this is of essence especially in larger specimens), the FHP conditions should equalize the non-uniformities due to the pressure-assisted mass transport in the densified specimen.

The thermal runaway occurs only at certain critical levels of the temperature (that is why initial heating by the external furnace is always necessary for most of the flash-sintering experiments) and applied voltage. Therefore, for the successful outcome of FHP the determination of the required critical values of these processing parameters is needed. Below the results of the preliminary analysis of the conditions of FHP of SiC powder are represented. Based on this analysis, the experiments on FHP of SiC powder, described in section on Methods, have been conducted.

Many ceramic materials demonstrate an exponential increase of electrical conductivity with temperature. According to ref. 39, electrical conductivity 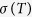 of SiC can be given as a function of temperature *T*:


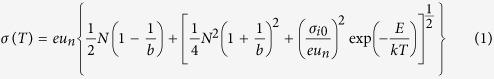


where 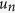 is the mobility of free electrons: 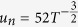, 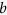 is the electron-hole mobility ratio taken equal to 5; N is the concentration of impurities estimated according to ref. 39 as 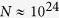; *E* is the SiC intrinsic conductivity activation energy, which is taken in our calculations to be equal to 3.1 eV; 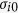 is equal to 8 · 10^5^; *k* is the Boltzmann constant. The function *σ(T*) is almost constant in the temperature interval between 1000 K and 1400 K. It is an interval of the SiC extrinsic conductivity depending predominantly on the impurity concentration. At the temperatures between 1400 K and 1500 K the intrinsic conductivity of SiC is initiated. At higher temperatures the intrinsic conductivity dominates, and the overall conductivity exponentially increases with temperature. In the case of a porous specimen, the conductivity decreases proportionally to the value of porosity. To take into account this effect, we use the modified Maxwell formula for the effective electrical conductivity of porous media^40^:


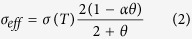


where *θ* is the porosity and fitting constant *α* is taken equal to 2, assuming zero conductivity for the initial free packing of particles with *θ* = 0.5.

According to Joule’s law, heat *Q* generated by electric current with a voltage *V* across the circuit is


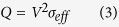


Heat *Q* increases temperature in the circuit. If conductivity increases with temperature like in the considered case of SiC ceramics, it promotes an increase of *Q* and, in turn, even more active temperature growth. As a result, in some cases a considerable acceleration of heating is observed and the heating rate can reach thousand degrees per second. This is how runaway heating or thermal runaway takes place.

**Figure 4:**
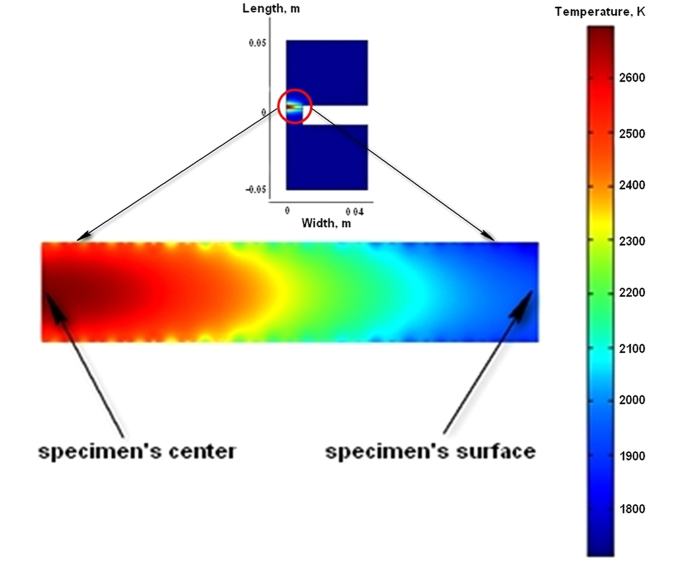
Temperature distribution after 2.4 s heating with 10 V electric current: (top) in the whole SPS set; (bottom) in the section of the coin-shaped specimen.

In the conducted preliminary modeling the temperature evolution of a coin-shaped SiC specimen placed between graphite punches is simulated. The considered geometry is clear from the Fig. 4. The thermo-electrical properties of graphite have been taken from ref. 41. The thermal properties of SiC correspond to ref. 42. The starting porosity of SiC specimen in the modeling is accepted to be *θ* = 0.3. The voltage *V* is assumed to be constant. The electrical conductivities of graphite and silicon carbide, as well as the thermal parameters of these materials are functions of temperature. At the outer surfaces of the punches and of the SiC specimen, the radiation cooling is assumed according to Stefan’s law. The starting temperature during modeling of heating has been taken equal to the melting temperature of copper (1357 K). All the calculations have been carried out using COMSOLTM finite element commercial software.

**Figure 5:**
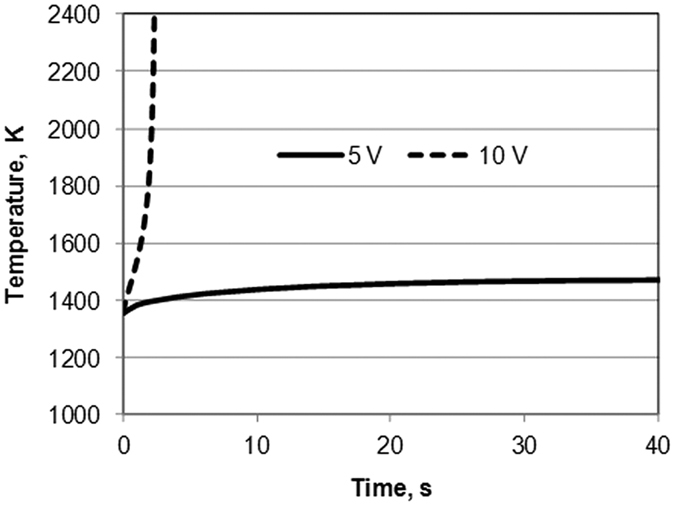
Temperature evolution for the voltage level 10 V (line 1) and 5 V (line 2).

The results of the modeling clearly demonstrate the threshold nature of the runaway heating. The evolution of temperatures for the voltage levels 5 V and 10 V are shown in Fig. 5. The calculations show that in the case of 10 V applied voltage the temperature level of 240 K is reached after about 2.4 seconds. The voltage level of 5 V allows only a small temperature increase about of 100 K for 60 seconds. The temperature distributions in the SPS setup for the 10 V applied voltage at the moment 2.4 s are shown in Fig. 5. The modeling predicts considerable temperature non-uniformity in the specimen because of a very high heating rate and low thermal conductivity of porous SiC.

As the modeling results indicate, the thermal runaway is triggered for SiC powder at the level of temperatures close to the melting point of copper. This renders the idea of utilizing a sacrificial copper collar (Figs 1, 2, 3). The main objective of the designed FHP setup is to provide external heating to the pre-consolidated SiC specimen (the SiC pellet was pre-densified by regular SPS to the level of 72% relative density) up to the level of temperature of about 1085 °C (melting point of copper) and then to make sure that the electrical current in the SPS system has only one way to pass – through the pre-heated SiC specimen (due to the melting, the copper collar removes itself from the contact with the upper electrode and, starting from that moment, the electric current cannot pass through it).

The rapid increase of the temperature in the developed FHP approach changes the conductivity of the initially practically non-conductive specimen so that the electric current can effectively pass through it – and this change is synchronized with establishing the contact between the upper electrode-punch and the specimen. Thereby the specimen becomes a part of the newly established electric circuit, which is not available in the beginning of the FHP process. This technical arrangement essentially distinguishes the FHP technique from regular SPS approaches involving intense heating of initially (from room temperature levels) conductive materials at the maximum power capacity of an SPS device, such as “flash spark plasma sintering” method^43^,^44^ applied to consolidate an initially electrically conductive ZrB_2_ specimen or to partially consolidate SiC specimen inside a permanently conductive layer of graphite felt.

The results of the conducted preliminary experiments on the FHP of SiC are very promising (Fig. 3). They indicate high degree of almost instantaneous densification with limited grain growth.

In connection with the results of the conducted experiments involving a special SPS tooling design, one can consider the future usage of hybrid heating SPS devices to be a much more powerful approach for the implementation of the flash spark plasma sintering concept (for which no special tooling design is needed). In fact, the employment of the SPS devices with the embedded possibility of an independent hybrid heating should be the mainstream direction for the future studies and practical utilization of the new flash hot pressing (ultra-rapid spark plasma sintering) technique.

## Methods

### Materials and Equipment Used

The sample powder used in all experiments is 1 micron sized, 99.9% pure SiC powder from Sigma Aldrich. Both pre-sintering and FHP were performed using a Dr. Sinter Lab Series spark plasma sintering machine (SPS Syntax Co.) Copper tubing used in the FHP die design is a common household plumbing pipe, 16.2 mm in diameter with a wall thickness of 0.5 mm. Support equipment includes various graphite dies and spacers, as well as graphite paper used in the pre-sintering process. Sample inspection was supported by FEI Quanta 450 FEG Scanning Electron Microscope. Samples for characterization were prepared using a Struers Tegra sample polishing system and QT150 Sputter Coating Machine with platinum target.

### Sample Preparation Procedures

**Figure 6:**
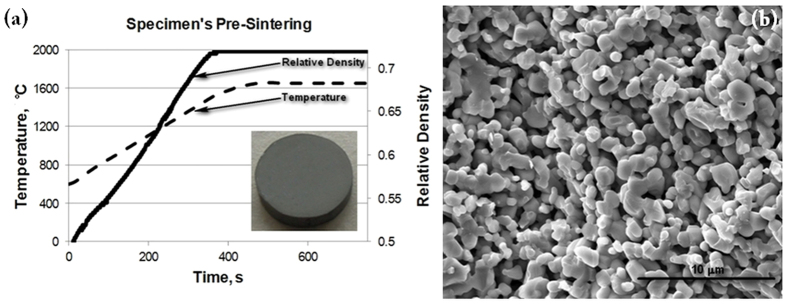
Pre-spark plasma sintering of the specimen to be subjected to FHP. The pre-sintered specimen’s relative density is 72%.

Before the flash sintering experiments were conducted, pre-sintered specimens had been prepared using the Spark-Plasma System Dr. Sinter 515S of the SPS Syntax Co. The pre-sintered compacts had to be used to provide the initial mechanical integrity to the processed powder volume before the moment of flash. The initial pre-sintered particle bonding among other factors should stabilize the conductive properties of the pre-sintered specimen. Graphite dies have been used to create a cylindrical specimen of low density with sufficient strength to withstand the stresses associated with handling of the specimen and other experiment-related preparations. To achieve this density, the following heating profile was used: 150 °C/min from 600 °C to 1500 °C, 100 °C/min from 1500 °C to 1600 °C, and 50 °C/min from 1600 °C to 1650 °C with a final holding time of 5 minutes. A typical heating profile is shown in Fig. 6(a). The pre-sintered specimen’s relative density is 72% (see Fig. 6(b)).

Once the pre-sintered specimen is consolidated, it is cleaned prior to flash sintering. Traces of the carbon paper that enveloped the specimen within the graphite die are removed from all surfaces with a Struers Tegra sample polishing device.

An example of a pre-sintered specimen is shown in Fig. 6(a). The specimen is allowed to air dry before its density is measured using the Archimedes principle. With this information recorded, the specimen is fully prepared for the FHP Experiment. This procedure was made consistent for all the experiments. The goal was to minimize variables in the pre-sintered specimen.

### Specimen Characterization Procedures

The first step in sample characterization is an accurate measurement of the specimen’s density. This is done using the Archimedes technique. This result then is compared to the theoretical density of the material in order to gauge the effectiveness with which the material was consolidated during sintering.

The second step in sample characterization is to look at the morphology of the specimen structure using scanning electron microscopy (SEM). This process can verify the level of densification achieved, and can be used to gauge the grain growth in the specimen. To prepare the specimen for SEM it is fractured to expose it’s inner structure and placed on a holder with graphite tape. The specimen is then coated with a sputtered platinum 6 nm thick layer.

### Control Samples

**Figure 7:**
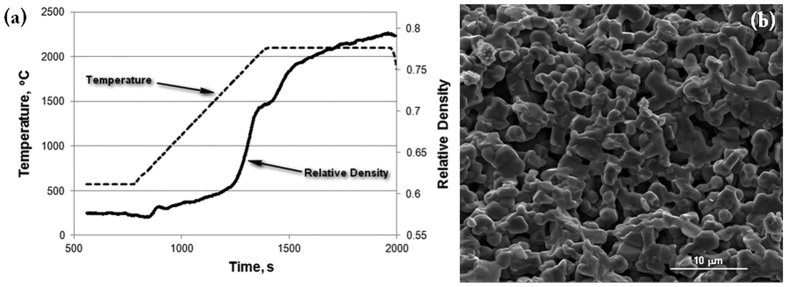
(**a**) The heating profile and shrinkage of SiC for a high temperature SPS process; (**b**) sample was sintered at 2100 °C using the SPS method. Note that there is neck formation between particles as well as grain growth. Despite the high temperature, densification is limited as can be seen by the presence of large pores.

As a means of judging the effectiveness of the FHP technique, various samples were produced with spark plasma sintering using the same SiC powder that was used in the flash sintering experiments. These samples were prepared at the extreme temperature of 2100 °C, as shown in Fig. 7, in order to give them the best opportunity to densify.

Characterization of the sample, shown in Fig. 7, shows that neck formation and grain growth did occur, but porosity of the sample remains high (about 83%). This is an example of how difficult it is to consolidate SiC materials. This sample is the standard by which the effectiveness of FHP can be judged. As shown by Grasso *et al.*^45^, spark plasma sintering under 2450 °C and pressure of 40MPa rendered specimens with the density of 93% (in some areas of the specimen’s volume only).

### FHP Procedure

Preparing the SPS device for FHP is not unlike the process for regular spark plasma sintering. Die spacers are used at either ends of the central die body to center the specimen between the top and bottom SPS contact plates. The top edge of the sacrificial copper collar is exposed to the radiation thermometer which is used by the SPS to regulate its temperature. As described above, the sacrificial collar is heated up to the melting temperature, at which point the top punch makes contact with the specimen, and a significant flash of light is usually observed immediately after.

Once the top punch has made contact with the specimen, the SPS device attempts to maintain the flow of the electric current through the die stack. In the conducted experiments this has resulted in a large spike on the analog voltage gauges present in the SPS devise. Every flash sintered specimen therefore has affectively almost no holding time.

With the experiment over, the SPS device chamber is allowed to cool and the specimen is removed from the die assembly. The specimen is then ready to be characterized.

### FHP Results

The FHP experiments have been conducted in accord with the tooling schematics described above (see Fig. 1). In the experiments we have utilized copper tubes of different heights. The hypothesis was that a taller tube would allow the specimen to be heated for a slightly longer period of time before it made electrical contact with the top punch. In such a case the conductivity of the SiC compact would be changed at the moment of the electrical contact with the top punch allowing for more current to flow through and potentially control the effectiveness of the FHP process.

**Figure 8:**
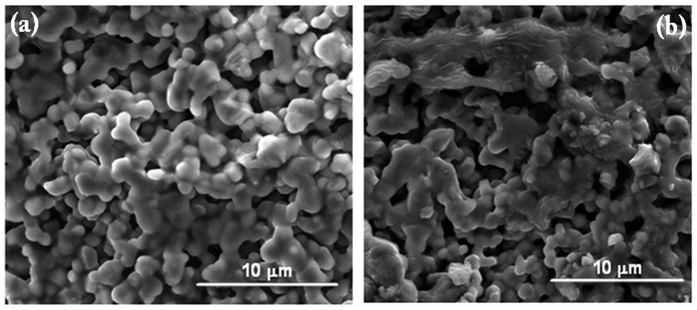
The impact of the height of the copper tube on the outcomes of the FHP experiments. For a taller tube height (**b**) a much higher level of sintering took place than the experiment with the shorter copper tube (**a**).

Indeed, the sample SEM images, Fig. 8, indicate the impact of the height of the copper tube on the outcomes of the FHP experiments. For a taller tube height (Fig. 8(b) – 86%) a much higher level of sintering took place than the experiment with the shorter copper tube (Fig. 8(a) – 77%). At the same time, a medium size height of the copper tube resulted in the best experimental outcomes shown in Fig. 2. The conducted tests clearly demonstrate that the height of the sacrificial collar can be used as a control factor influencing the degree of powder consolidation under FHP conditions. It is evident that the material of the sacrificial collar can also play the role of the factor controlling the starting temperature of the thermal runaway and, thus, influence the outcomes of the consolidation process.

**Figure 9:**
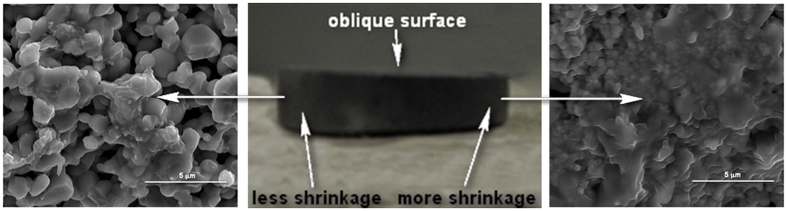
Experiments on FHP using a punch with an oblique surface (**b**). The results of the experiments indicate a clear difference in the degree of the consolidation of SiC powder specimen in the areas subjected to the different levels of pressure (**a**,**c**).

When pressed by a regular punch, the obtained highly densified specimens have mostly uniform structure (Fig. 2(b)) with some small pilled-off areas at the edges. Specimens with a lesser degree of densification show some clustered densified and porous areas (Fig. 8). Specimens pressed by a sloped punch, as indicated by the experiments described below (Fig. 9) show the structure with a gradient distribution of relative density across the punch radial direction.

Additional series of experiments on FHP have been conducted using a punch with an oblique surface (Fig. 9). This punch shape was utilized to check the effect of the pressure on the mass transport, and ultimately on the densification under FHP conditions. The results of the experiments indicate a clear difference in the degree of the consolidation of the SiC powder specimen in the areas subjected to the different levels of pressure (Fig. 9(a) – 78% and (c) – 91%). This experimental result provides an indirect confirmation of a possible densification equalizing effect of the (properly controlled) external pressure during FHP as opposed to free flash sintering – a factor potentially favoring the FHP process scalability.

## Additional Information

**How to cite this article**: Olevsky, E. A. *et al.* Flash (Ultra-Rapid) Spark-Plasma Sintering of Silicon Carbide. *Sci. Rep.* **6**, 33408; doi: 10.1038/srep33408 (2016).
